# Action Steps and Strategies Taken by Older Persons to Prevent and Mitigate Effects of Climate Change

**DOI:** 10.1093/geroni/igab046.2261

**Published:** 2021-12-17

**Authors:** Martha Bial

**Affiliations:** Fordham University, West Harrison, New York, United States

## Abstract

While the vulnerability of older persons to climate change is recognized by many scholars, there has been less attention to contributions older adults make to the fight against climate change, and their motivations to engage in that fight. Motivations include concern for the environment they will leave to their descendants. Contributions include freed up time, and skills gained in personal or work history to educate others on the issues and to organize and advocate for policy change. This presentation will highlight several national and international organizations of older people devoted to educating community groups, monitoring water quality and changes in wildlife habitats, and testifying before legislative bodies in campaigns for increased environmental regulation. Some of these organizations are intergenerational, providing additional benefits in cross-generational social exchange. Such activities are in line with SDGs 13 (action on climate change), 11 (sustainable cities), 12 (sustainable consumption) and 17 (expanding multi-stakeholder partnerships).

